# Supplementary material for: Mortality caused by tropical cyclones in the United States
Source: Nature. 2024 Oct 2;635(8037):121–8. doi: 10.1038/s41586-024-07945-5 (PMC11541193; doi:10.1038/s41586-024-07945-5)
Supplement: Supplementary file 1 — Supplementary Figs. 1–7. [file 41586_2024_7945_MOESM1_ESM.pdf]

nature portfolio

https://doi.org/10.1038/s41586-024-07945-5

Supplementary information

Mortality caused by tropical cyclones in the United States

In the format provided by the authors and unedited

Supplemental Information

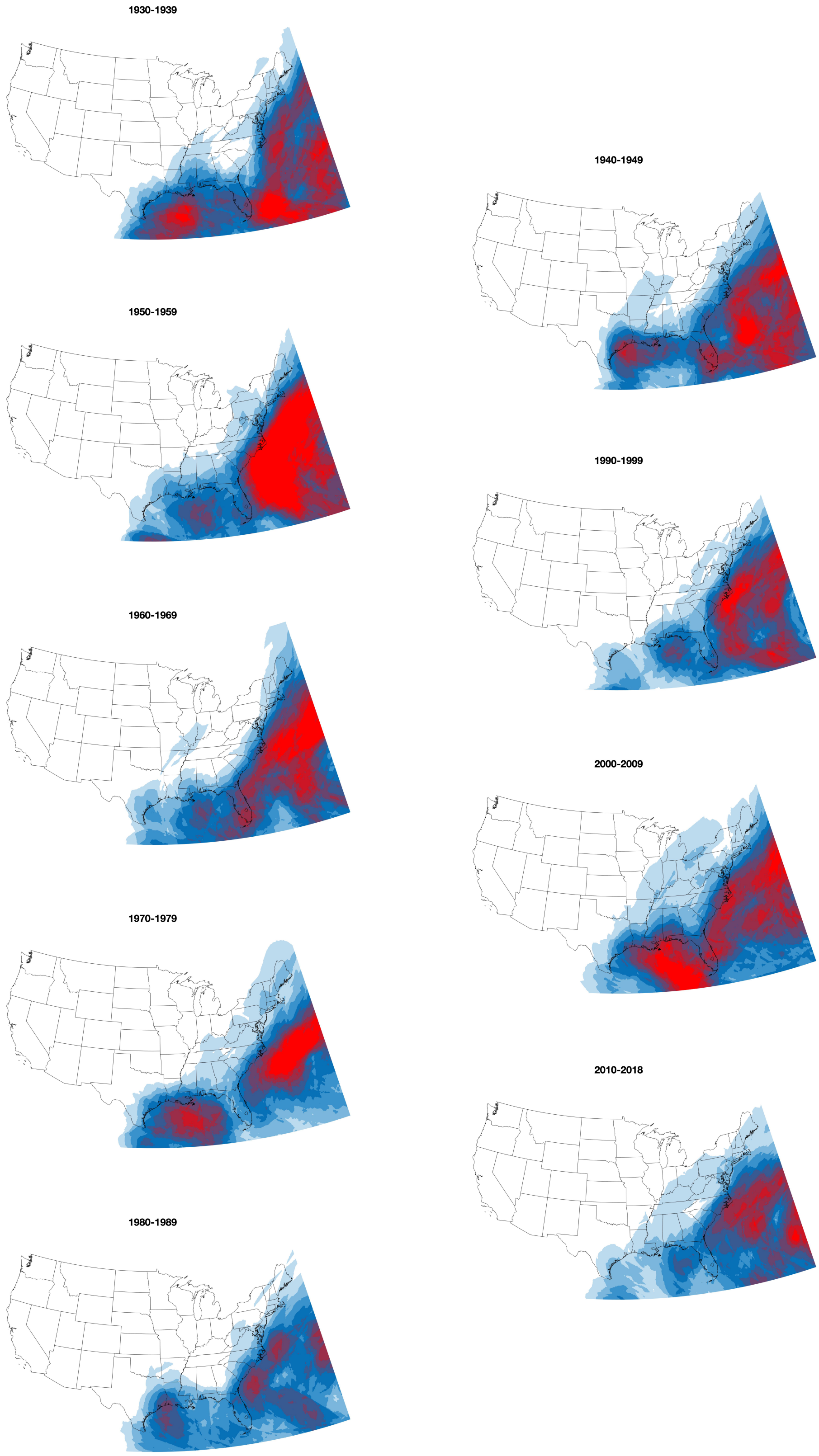

Figure 1: **Tropical cyclone incidence data.** Limited Information Cyclone Reconstruction and Integration for Climate and Economics (LICRICE) modeled cumulative maximum wind speed per decade from tropical cyclones at each $0.1° \times 0.1°$ pixel, between 1930 and 2018. Red = 500 ms^{-1} (maximum) and dark blue = 50 ms^{-1} (minimum). Econometric analysis is implemented using monthly values.

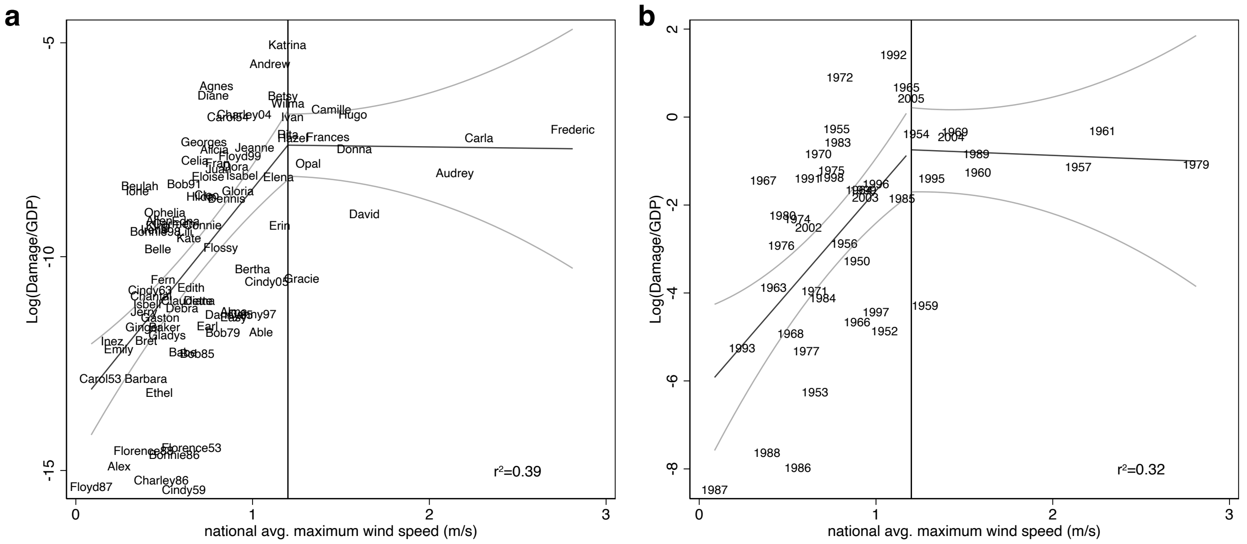

Figure 2: **Comparison of tropical cyclone damages vs wind speed incidence.** (**a**) Total logarithm tropical cyclone national economic damage per national gross domestic product (GDP), assembled for Nordhaus 2010,[1] of tropical cyclones between 1950-2005 and national average maximum wind speed (ms^{-1}) estimated from LICRICE. Discrete spline estimated relationship, split at 1.2 maximum wind speed (ms^{-1}). R-squared = 0.39. (**b**) corresponding annual tropical cyclone log damage per GDP and national average maximum wind speed (ms^{-1}). R-squared = 0.32.

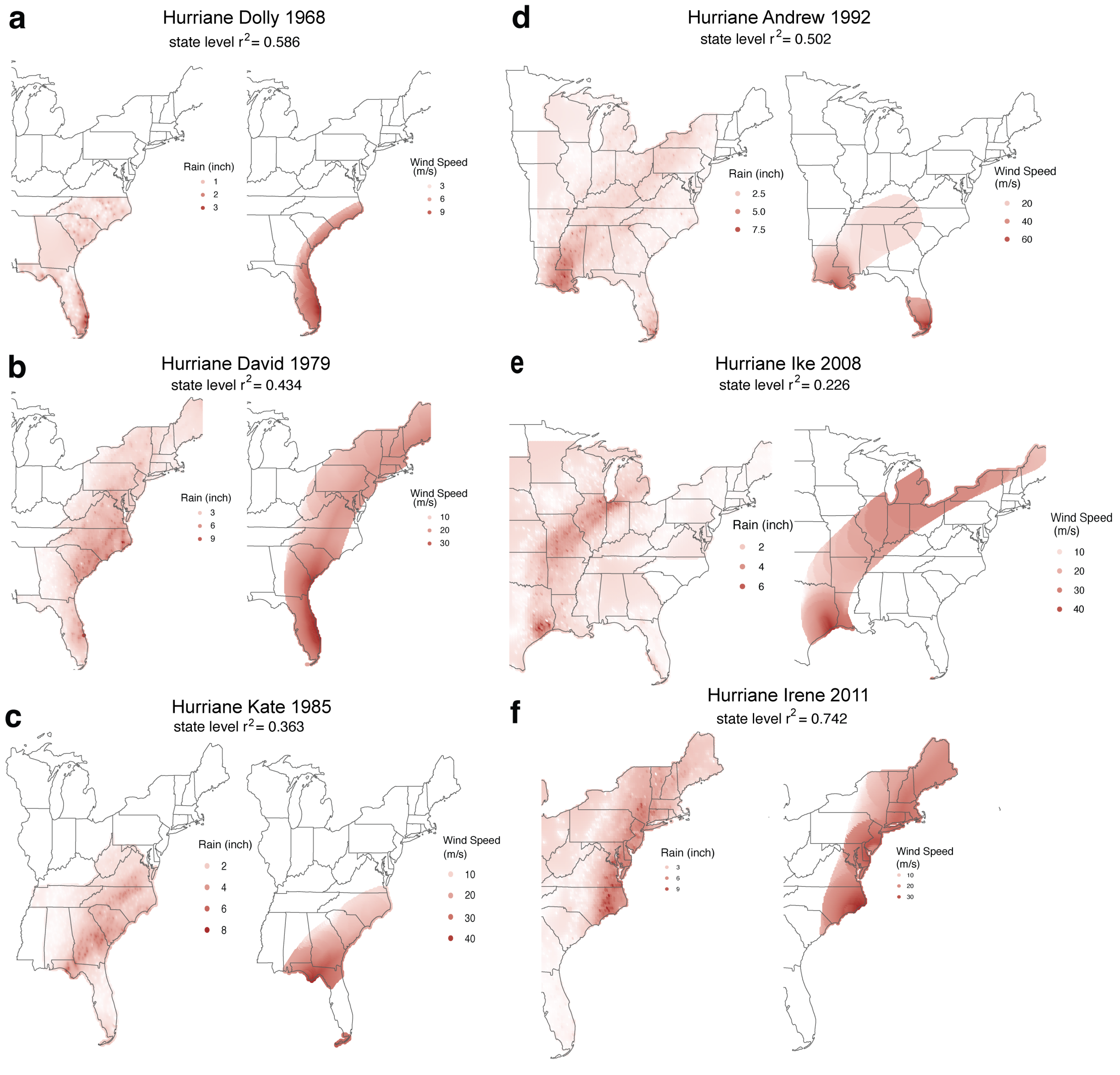

Figure 3: **Example of tropical cyclone total rainfall and maximum wind speeds.** Left panel, NCEP tropical cyclone rainfall weather station data (inches) inverse squared distance weighted interpolated at each $1° \times 1°$ pixel. Right panels, LICRICE modeled cumulative maximum wind speed (ms^{-1}) from tropical cyclones at each $0.1° \times 0.1°$ pixel. R-squared values from the linear correlation between state area average rainfall and state maximum wind speed for each storm.

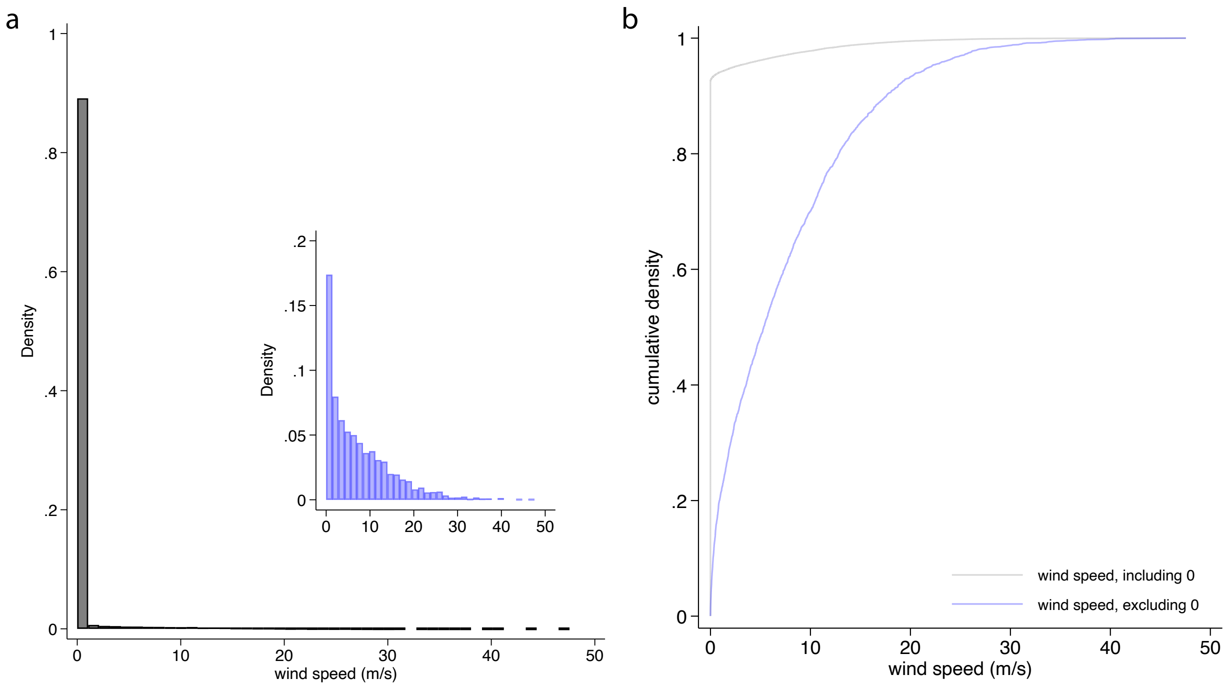

Figure 4: **Probability density and cumulative probability of state monthly maximum wind speed from tropical cyclones.** (**a**) density plots of the LICRICE modeled monthly maximum wind speed from tropical cyclones between 1950 and 2015 for 36 states in sample. Blue plot excludes observations with wind speed incidence equal to zero. (**b**) Cumulative density plots of the monthly maximum wind speed from tropical cyclones. Blue plot excludes observations with wind speed incidence equal to zero.

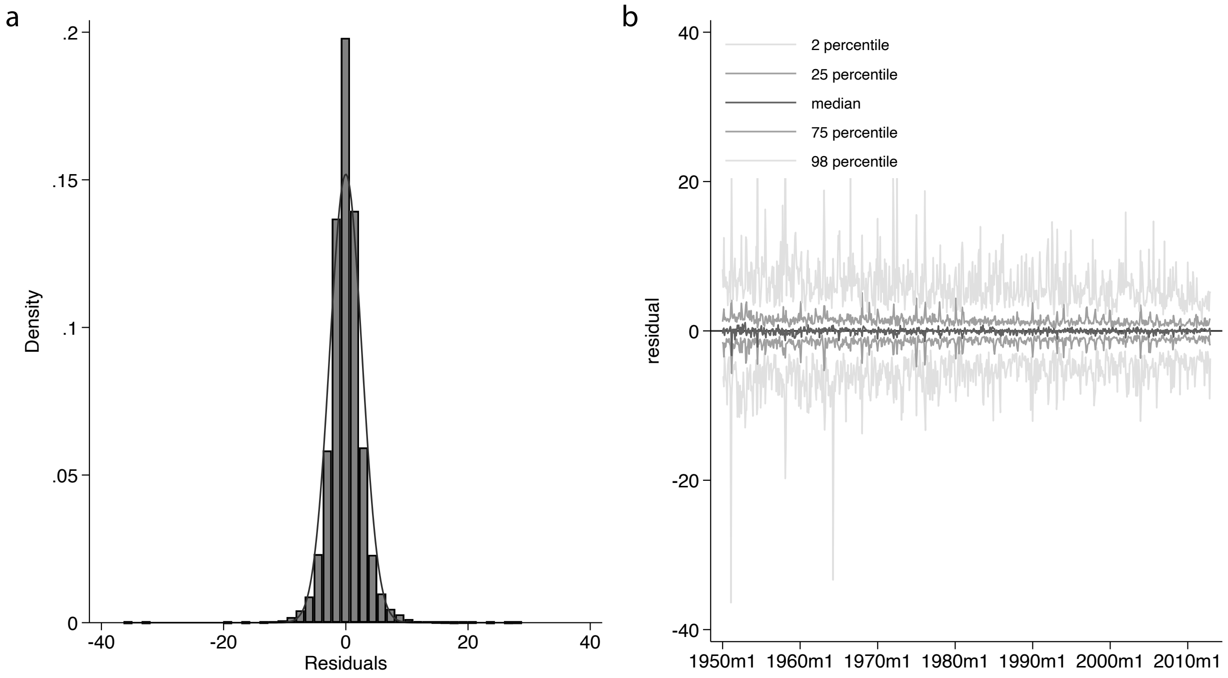

Figure 5: **Distribution of the residuals from the main linear model.** (**a**) Density plot of the residuals from the impulse response of mortality rates following state-level wind speed incidence. Gray line = normal distribution. (**b**) Percentiles of residuals across each month between 1950 - 2015.

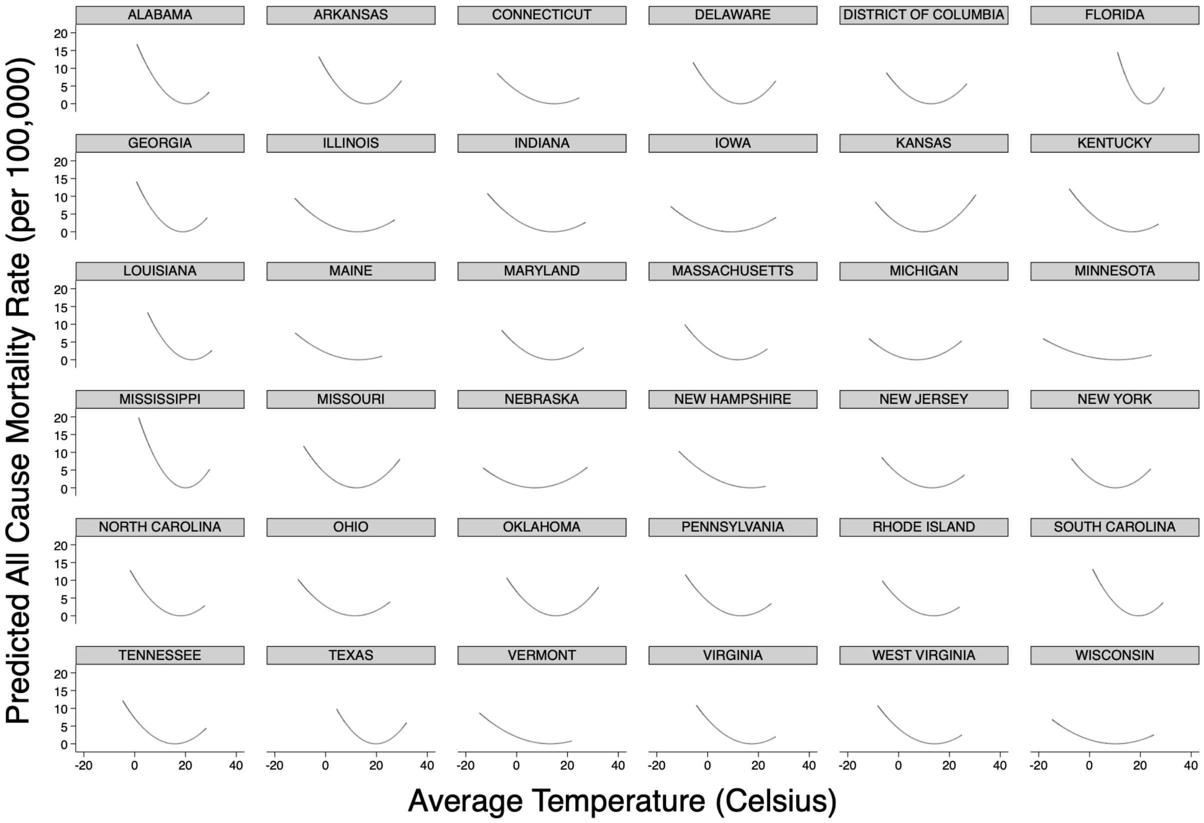

Figure 6: **Influence of temperature on mortality by state.** Estimated state-specific temperature-mortality responses. Quadratic lines of temperature (Celsius) and predicted all-cause mortality rate (per 100,000).

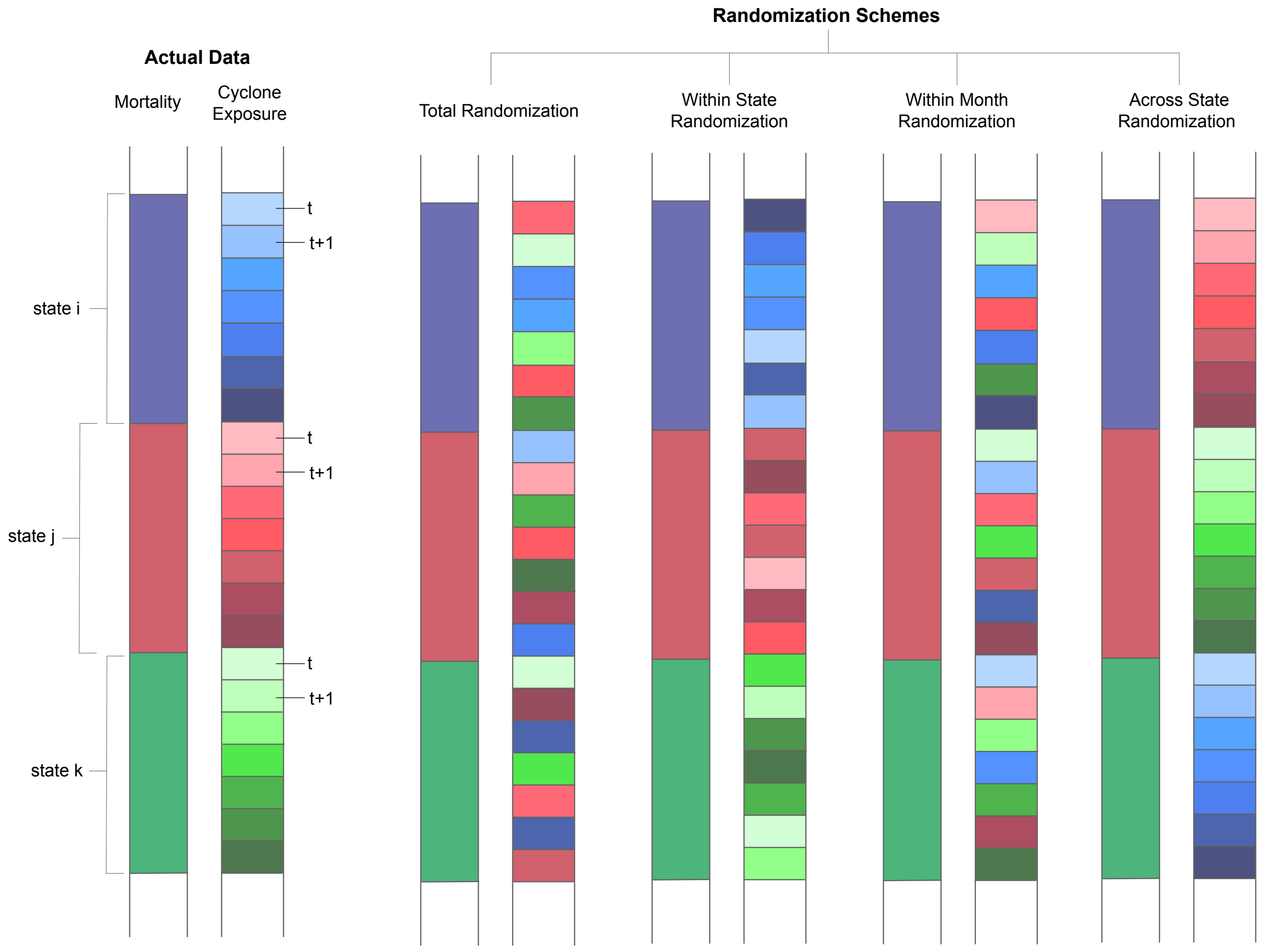

Figure 7: **Illustration of the randomization-based placebo tests.** Blue, red and green boxes (Actual Data: Mortality) represent mortality for state $i - k$. Color gradients (Actual Data: Cyclone Exposure) represents a timing sequence of cyclone incidence (t, $t+1$, $t+2$,...). Randomization Schemes, Entire Sample (total) = "Total Randomization" (in methods), with color and color sequence shuffled; Across State = "Across State Randomization" with color shuffled but color sequence fixed; Within State = "Within State Randomization" with colors fixed but color sequence shuffled; and Within Month = "Within Month Randomization" with color sequence fixed but colors shuffled. Illustration adapted from Hsiang & Jina 2014.[2]

References

[1] W. D. Nordhaus, "The economics of hurricanes in the United States," Working Paper 12813, National Bureau of Economic Research, December 2006.

[2] S. M. Hsiang and A. S. Jina, "The causal effect of environmental catastrophe on long-run economic growth: Evidence from 6,700 cyclones," Working Paper 20352, National Bureau of Economic Research, July 2014.
