# Supplementary material for: Mortality caused by tropical cyclones in the United States
Source: Nature. 2024 Oct 2;635(8037):121–8. doi: 10.1038/s41586-024-07945-5 (PMC11541193; doi:10.1038/s41586-024-07945-5)
Supplement: Supplementary file 2 — Reporting Summary [file 41586_2024_7945_MOESM2_ESM.pdf]

nature portfolio

Corresponding author(s): Solomon Hsiang
Last updated by author(s): Dec 27, 2023

# Reporting Summary

Nature Portfolio wishes to improve the reproducibility of the work that we publish. This form provides structure for consistency and transparency in reporting. For further information on Nature Portfolio policies, see our Editorial Policies and the Editorial Policy Checklist.

## Statistics

For all statistical analyses, confirm that the following items are present in the figure legend, table legend, main text, or Methods section.

| n/a | Confirmed | |
|---|---|---|
| ☐ | ☒ | The exact sample size (*n*) for each experimental group/condition, given as a discrete number and unit of measurement |
| ☐ | ☒ | A statement on whether measurements were taken from distinct samples or whether the same sample was measured repeatedly |
| ☐ | ☒ | The statistical test(s) used AND whether they are one- or two-sided<br>*Only common tests should be described solely by name; describe more complex techniques in the Methods section.* |
| ☐ | ☒ | A description of all covariates tested |
| ☐ | ☒ | A description of any assumptions or corrections, such as tests of normality and adjustment for multiple comparisons |
| ☐ | ☒ | A full description of the statistical parameters including central tendency (e.g. means) or other basic estimates (e.g. regression coefficient) AND variation (e.g. standard deviation) or associated estimates of uncertainty (e.g. confidence intervals) |
| ☐ | ☒ | For null hypothesis testing, the test statistic (e.g. *F*, *t*, *r*) with confidence intervals, effect sizes, degrees of freedom and *P* value noted<br>*Give P values as exact values whenever suitable.* |
| ☒ | ☐ | For Bayesian analysis, information on the choice of priors and Markov chain Monte Carlo settings |
| ☐ | ☒ | For hierarchical and complex designs, identification of the appropriate level for tests and full reporting of outcomes |
| ☐ | ☒ | Estimates of effect sizes (e.g. Cohen's *d*, Pearson's *r*), indicating how they were calculated |

*Our web collection on statistics for biologists contains articles on many of the points above.*

## Software and code

Policy information about availability of computer code

| | |
|---|---|
| Data collection | Code was not used to collect data for the study. |
| Data analysis | Stata 14, Matlab 2019a, R 4.2.0 |

For manuscripts utilizing custom algorithms or software that are central to the research but not yet described in published literature, software must be made available to editors and reviewers. We strongly encourage code deposition in a community repository (e.g. GitHub). See the Nature Portfolio guidelines for submitting code & software for further information.

## Data

Policy information about availability of data

All manuscripts must include a data availability statement. This statement should provide the following information, where applicable:

- Accession codes, unique identifiers, or web links for publicly available datasets
- A description of any restrictions on data availability
- For clinical datasets or third party data, please ensure that the statement adheres to our policy

All data is available for download here: https://zenodo.org/uploads/10459719.

The full data processing code is not included but the collected full data set needed for the main analysis (figures 1-4) is provided in DATA_hurricane_mortality_temp_month_state_19302015.dta }. This includes the matched LICRICE generated TC wind speed and pddi; the all-cause mortality data

from the Center for Disease Control and Prevention (CDC) Mortality Statistics of the United States annual volumes, the Multiple Cause of Death (MCOD) files, and Underlying Cause of Death database; the population data from the Inter-university Consortium for Political and Social Research and the US Census Bureau, Intercensal Population and Housing Unit Estimates; and the temperature data from the Berkeley Earth Surface Temperatures (BEST).

We also provide the follow datasets required for the analysis:

- Shapefiles of the U.S. states : cb_2016_us_state_20m
- LICRICE generated TC wind speed and pddi by state and month : panel_by_storm__NA_USA_density_8_yr_1930_2018.csv
- NOAA TC direct deaths : directdeaths.csv
- Nordhaus TC damages and LICRICE, national : nordhaus_LICRICE_USA_merged.dta
- TC rainfall data : rainfall_idw_state_storm.csv
- CDC mortality data for all states : mortality_19002015.dta (needed for SI figures)
- Counties on the coastline : coastline-counties-list.xlsx (needed for SI figures)
- County population by age : us.1969_2020.19ages.adjusted.txt (needed for SI figures)
- List of TC names : storm_list.txt
- Wind speed and population by pixel : wind_state_pop_export.csv (needed for Figure SI13e)
- LICRICE all storms pixel-level : NA_USA_density_8_yr_1930_2018_storm_specific.mat (needed for Figure SI6)
- Shapefile of US states : plotting_maps/s_11au16/s_11au16.shp (needed for Figure SI1)
- Hurricane direct death data : directdeaths.csv (needed for Figure 3)
- TC unique serial number and storm name : storm_id_name_raw.mat (needed for Figure 3)
- TC unique serial number : stormnamelist.mat (needed for Figure 3)

## Research involving human participants, their data, or biological material

Policy information about studies with human participants or human data. See also policy information about sex, gender (identity/presentation), and sexual orientation and race, ethnicity and racism.

| | |
|---|---|
| Reporting on sex and gender | Information on sex and gender were unavailable for analysis. |
| Reporting on race, ethnicity, or other socially relevant groupings | In one of our analyses, we stratify the sample based on whether individuals are identified as "Black" or "White", excluding other races, because these two categories are the only consistent categories available in our sample. These are recorded in administrative data maintained by the US Centers for Disease Control. |
| Population characteristics | See above |
| Recruitment | No recruitment was done for the study |
| Ethics oversight | We received a waiver from the UC Berkeley IRB |

Note that full information on the approval of the study protocol must also be provided in the manuscript.

# Field-specific reporting

Please select the one below that is the best fit for your research. If you are not sure, read the appropriate sections before making your selection.

☐ Life sciences ☒ Behavioural & social sciences ☐ Ecological, evolutionary & environmental sciences

For a reference copy of the document with all sections, see nature.com/documents/nr-reporting-summary-flat.pdf

# Behavioural & social sciences study design

All studies must disclose on these points even when the disclosure is negative.

| | |
|---|---|
| Study description | Quantitative analysis of longitudinal administrative data combined with reconstructions of physical geophysical events. |
| Research sample | Sample is representative of the contiguous United States because it includes all individuals living in the contiguous United States during 1930-2015. We chose this study sample because of data availability. Detailed all-cause mortality data is not available for this sample period outside of the United States. All cause mortality data was collected from the Center for Disease Control and Prevention (CDC) Mortality Statistics of the United States annual volumes, the Multiple Cause of Death (MCOD) files, and Underlying Cause of Death database (2017). State population data was combined from Inter-university Consortium for Political and Social Research and the US Census Bureau, Intercensal Population and Housing Unit Estimates. |
| Sampling strategy | Our sample is comprehensive administrative data, representing the universe of recorded deaths in the contiguous United States during the period of study. We included data from 1930-2015 because it was all of the publicly available digitized data. This long time period allows us to estimate the lagged effects for 20 years after a TC. |
| Data collection | Administrative data on all-cause mortality and population is collected by local, state, and federal government agencies. The data collection did not involved experimental conditions or a study hypothesis. |

| | |
|---|---|
| Timing | Monthly data during 1930-2015. |
| Data exclusions | No data is excluded. |
| Non-participation | No participants were involved in the study. |
| Randomization | The research design assumes that the timing and intensity of tropical cyclone incidence within a given location is as good as randomly assigned to populations. All quasi-experimental comparisons are within a population over time and outcomes are not compared across locations. |

# Reporting for specific materials, systems and methods

We require information from authors about some types of materials, experimental systems and methods used in many studies. Here, indicate whether each material, system or method listed is relevant to your study. If you are not sure if a list item applies to your research, read the appropriate section before selecting a response.

## Materials & experimental systems

| n/a | Involved in the study |
|---|---|
| ☒ | ☐ Antibodies |
| ☒ | ☐ Eukaryotic cell lines |
| ☒ | ☐ Palaeontology and archaeology |
| ☒ | ☐ Animals and other organisms |
| ☒ | ☐ Clinical data |
| ☒ | ☐ Dual use research of concern |
| ☒ | ☐ Plants |

## Methods

| n/a | Involved in the study |
|---|---|
| ☒ | ☐ ChIP-seq |
| ☒ | ☐ Flow cytometry |
| ☒ | ☐ MRI-based neuroimaging |

## Plants

| | |
|---|---|
| Seed stocks | n/a |
| Novel plant genotypes | n/a |
| Authentication | n/a |
